# Supplementary material for: Disparities in distribution of COVID-19 vaccines across US counties: A geographic information system–based cross-sectional study
Source: PLoS Med. 2022 Jul 28;19(7):e1004069. doi: 10.1371/journal.pmed.1004069 (PMC9333439; doi:10.1371/journal.pmed.1004069)
Supplement: S1 STROBE Checklist — (DOC) [file pmed.1004069.s001.doc]

STROBE Statement—Checklist of items that should be included in reports of ***cross-sectional studies***

|  | Item No | Recommendation |
| --- | --- | --- |
| **Title and abstract** | 1 | (*a*) Indicate the study’s design with a commonly used term in the title or the abstract  Title indicates study design |
|  |  | (*b*) Provide in the abstract an informative and balanced summary of what was done and what was found  Abstract |
| Introduction | | |
| Background/rationale | 2 | Explain the scientific background and rationale for the investigation being reported  Introduction, paragraphs 1 and 2. |
| Objectives | 3 | State specific objectives, including any prespecified hypotheses  Introduction, paragraph 3. |
| Methods | | |
| Study design | 4 | Present key elements of study design early in the paper  Methods section, data sources subsection |
| Setting | 5 | Describe the setting, locations, and relevant dates, including periods of recruitment, exposure, follow-up, and data collection  Data sources are listed in the methods section, data sources subsection |
| Participants | 6 | (*a*) Give the eligibility criteria, and the sources and methods of selection of participants  The observations were facilities, not participants, the types of facilities included are listed in the first paragraph of the methods section, data sources subsection |
| Variables | 7 | Clearly define all outcomes, exposures, predictors, potential confounders, and effect modifiers. Give diagnostic criteria, if applicable  Methods section, outcome and independent variables subsections |
| Data sources/ measurement | 8* | For each variable of interest, give sources of data and details of methods of assessment (measurement). Describe comparability of assessment methods if there is more than one group  Methods section, outcome and independent variables subsections |
| Bias | 9 | Describe any efforts to address potential sources of bias  Not applicable |
| Study size | 10 | Explain how the study size was arrived at  Not applicable – no study participants are involved in the study. Numbers of facilities included are reported in the first paragraph of the results section. |
| Quantitative variables | 11 | Explain how quantitative variables were handled in the analyses. If applicable, describe which groupings were chosen and why  Methods section, statistical analysis subsection |
| Statistical methods | 12 | (*a*) Describe all statistical methods, including those used to control for confounding  Methods section, statistical analysis subsection |
|  |  | (*b*) Describe any methods used to examine subgroups and interactions  Methods section, statistical analysis subsection |
|  |  | (*c*) Explain how missing data were addressed  Not applicable |
|  |  | (*d*) If applicable, describe analytical methods taking account of sampling strategy  Not applicable |
|  |  | (*e*) Describe any sensitivity analyses  Not applicable |
| Results | | |
| Participants | 13* | (a) Report numbers of individuals at each stage of study—eg numbers potentially eligible, examined for eligibility, confirmed eligible, included in the study, completing follow-up, and analysed  Numbers of facilities included are reported in the first paragraph of the results section |
|  |  | (b) Give reasons for non-participation at each stage  Not applicable – no study participants are involved in the study |
|  |  | (c) Consider use of a flow diagram  Not applicable – no study participants are involved in the study |
| Descriptive data | 14* | (a) Give characteristics of study participants (eg demographic, clinical, social) and information on exposures and potential confounders  Not applicable – no study participants are involved in the study. |
|  |  | (b) Indicate number of participants with missing data for each variable of interest  Not applicable – no study participants are involved in the study |
| Outcome data | 15* | Report numbers of outcome events or summary measures  First paragraph of results section and Table |
| Main results | 16 | (*a*) Give unadjusted estimates and, if applicable, confounder-adjusted estimates and their precision (eg, 95% confidence interval). Make clear which confounders were adjusted for and why they were included  Paragraphs 1-4 of results section |
|  |  | (*b*) Report category boundaries when continuous variables were categorized  Paragraphs 1-4 of results section |
|  |  | (*c*) If relevant, consider translating estimates of relative risk into absolute risk for a meaningful time period  Not applicable, cross sectional study. |
| Other analyses | 17 | Report other analyses done—eg analyses of subgroups and interactions, and sensitivity analyses  Second paragraph of results section. |
| Discussion | | |
| Key results | 18 | Summarise key results with reference to study objectives  First paragraph of discussion section |
| Limitations | 19 | Discuss limitations of the study, taking into account sources of potential bias or imprecision. Discuss both direction and magnitude of any potential bias  Paragraphs 3-4 of discussion section |
| Interpretation | 20 | Give a cautious overall interpretation of results considering objectives, limitations, multiplicity of analyses, results from similar studies, and other relevant evidence  Second paragraph of discussion section |
| Generalisability | 21 | Discuss the generalisability (external validity) of the study results  Third paragraph of discussion section |
| Other information | | |
| Funding | 22 | Give the source of funding and the role of the funders for the present study and, if applicable, for the original study on which the present article is based  Reported per PLOS Medicine guidelines |

*Give information separately for exposed and unexposed groups.

**Note:** An Explanation and Elaboration article discusses each checklist item and gives methodological background and published examples of transparent reporting. The STROBE checklist is best used in conjunction with this article (freely available on the Web sites of PLoS Medicine at http://www.plosmedicine.org/, Annals of Internal Medicine at http://www.annals.org/, and Epidemiology at http://www.epidem.com/). Information on the STROBE Initiative is available at www.strobe-statement.org.
