# Supplementary material for: Disparities in distribution of COVID-19 vaccines across US counties: A geographic information system–based cross-sectional study
Source: PLoS Med. 2022 Jul 28;19(7):e1004069. doi: 10.1371/journal.pmed.1004069 (PMC9333439; doi:10.1371/journal.pmed.1004069)
Supplement: Attachment — Submitted filename: Review PLOS Medicine_2022 COVID vaccine distribution inequities.docx [file pmed.1004069.s002.docx]

Disparities in Distribution of COVID-19 vaccines across US Counties

Hernandez et al.

The authors present a study of COVID-19 vaccination sites, with the goal of evaluating fairness of vaccine access.

Major comments

Introduction

Please include a summary of or at least references to one of the many frameworks that were created prior to COVID-19 vaccine availability to support equitable distribution of vaccines, e.g., PMID: 3237895. In the discussion section, consider commenting upon our success at following the goals laid out in one or more of these frameworks. Additionally, it’s important to recall that the US allowed COVID-19 vaccine eligibility in phases; this should be mentioned in the introduction as it likely affected placement of vaccine sites.

The authors state, “to our knowledge, no nationwide studies have measured health equity in the actual distribution of COVID-19 vaccines to healthcare facilities;” however, studies taking a different approach to evaluation of health equity in vaccine distribution have been published and are easily accessible, e.g., PMID: 34213561. The authors should consider a more thorough literature review to provide the reader with a succinct summary of the work that has already been done on health equity and vaccine distribution in the US.

Methods and Results

As this study only evaluated vaccination sites in place as of May 2021, it’s important to consider how eligibility for and availability of vaccines changed over time (<https://www.cdc.gov/vaccines/imz-managers/downloads/Covid-19-Vaccination-Program-Interim_Playbook.pdf>, see page 12), and to note that this varied by county. If possible, it would be helpful for the authors to compare their data to similar data later in 2021 or in early 2022. If this is not possible, the study should be clearly characterized as an early evaluation of vaccine sites, e.g., in the first sentence of the Results section should read, “…provided COVID-19 vaccinations as of May 2021.”

Consider showing the outcome(s)/independent variables stratified by the population types that were eligible for vaccination during their study period, which ended in May 2021 (therefore, only 3-4 months after COVID-19 vaccines were available to only certain portions of the population [e.g., healthcare workers who likely were vaccinated at their places of employment, and the elderly, who were likely vaccinated at their physician’s office). In other words, during the time when vaccines were only available for healthcare workers and people >65, were vaccines easily accessible to the corresponding elderly portion of the population?

Additionally, it would be helpful for the authors to comment upon the dates of their independent variable data. E.g., did they obtain the COVID-19 mortality data from the Johns Hopkins dashboard in or before May 2021 so that it temporally corresponded with their vaccine site data?

The table suggests that proportions of vaccine sites available to counties with varying levels of Black/Hispanic population did not vary significantly (e.g., for counties with black population <12.5%, 12.5%-42.2%, and >42.2%, the percent of healthcare facilities that served as vaccine sites was 60.5%, 63.9%, and 55.5%, respectively. It would be helpful for the authors to include a statistical comparison of the differences in these proportions and comment upon any significant findings, rather than merely mentioning the proportions of select groups in the Results section.

Discussion

Again, the authors’ results should be characterized as “early” if they are unable to show later analyses for comparison. E.g., in the first sentence of the Discussion, they should write “...disparities in the early distribution…”

Consider offering suggestions as to why the findings may be true. E.g., what barriers challenged availability of COVID-19 vaccination centers in the “hardest hit” communities? Were early COVID-19 vaccination sites targeted at locations with concentrated populations of people >65yo regardless of race because only people >65yo were eligible for vaccination at the time of the study?

It doesn’t make sense to evaluate availability/accessibility of vaccine sites when most of the county population was not eligible for vaccination. For most of the study period, which ended in May 2021, only a relatively small proportion of the population was eligible in most counties (e.g., healthcare workers, the elderly, people with certain medical conditions). I think it’s important that the authors discuss (and hopefully address by updating their data/analysis!) these limitations in the discussion section. In the last paragraph of the Discussion they provide a call to action that may not be necessary if vaccine equity did in fact improve after the study period. Ideally the authors would apply the same methods to evaluate health equity at vaccine sites at a later period as well, e.g., in late 2021 and/or early 2022.
