# Supplementary material for: Disparities in distribution of COVID-19 vaccines across US counties: A geographic information system–based cross-sectional study
Source: PLoS Med. 2022 Jul 28;19(7):e1004069. doi: 10.1371/journal.pmed.1004069 (PMC9333439; doi:10.1371/journal.pmed.1004069)
Supplement: Attachment — Submitted filename: response letter_3-30-2022.doc [file pmed.1004069.s003.doc]

**Requests from the editors:**

1) Please revise your title according to PLOS Medicine's style. Your title must be nondeclarative and not a question. It should begin with main concept if possible. Please place the study design in the subtitle (i.e., after a colon), e.g., a retrospective cohort study.

We have updated the title accordingly and included the study design as instructed: “Disparities in Distribution of COVID-19 Vaccines across U.S. Counties: A Geographic Information System-Based Cross-sectional Study”.

2)	Please include line numbers in your next draft.

We have added line numbers.

3) Is there a chance you can obtain more recent data from this setting?

Unfortunately, we are not able to update our results with more recent data. However, we believe our data is of main relevance because it represents the early distribution of COVID-19 vaccines in May 2021, when the general public first became eligible for vaccination (President Biden required all states to make vaccines available to all adults by May 1 2021). [1]

We have acknowledged that our findings represent the early distribution of COVID-19 vaccines in the abstract and repeatedly in the discussion section. We have also stated that future studies are necessary to test whether disparities persisted over time:

- “Our analyses did not include non-traditional vaccination sites and are based on data as of May 2021, thus they represent the early distribution of COVID-19 vaccines” (abstract results);
- “Healthcare facilities in counties with higher Black composition, in rural areas, and in hardest-hit communities were less likely to serve as COVID-19 vaccine administration locations in early 2021” (abstract conclusion);
- “To identify actual COVID-19 vaccine administration locations, we extracted VaccineFinder data from the Centers for Disease Control and Prevention (CDC) as of May 10, 2021. These data capture the healthcare facilities involved in the early distribution of COVID-19 vaccines, when the general public became eligible for vaccination nationwide” (methods);
- “To our knowledge, we present the first nationwide study to quantify disparities in the early distribution of COVID-19 vaccines to healthcare facilities” (discussion first paragraph);
- “Our analyses are based on data from May 2021, and thus represent the early distribution of COVID-19 vaccines, when every US adult became eligible for vaccination. Future studies should reproduce analyses to test whether disparities have persisted over time” (discussion third paragraph);
- “We evaluated whether the likelihood of healthcare facilities serving as COVID-19 vaccine administration sites in the early distribution of COVID-19 vaccines varied with the sociodemographic composition of the population served” (discussion conclusion).

4)	Abstract:

a)	Please ensure that all numbers presented in the abstract are present and identical to numbers presented in the main manuscript text.

The numbers in the abstract are identical to those in the main text.

b)	Please quantify the main results (please present both 95% CIs and p values).

Results have been updated accordingly.

c)	In the last sentence of the Abstract Methods and Findings section, please describe the main limitation(s) of the study's methodology.

We have added a sentence as indicated to note the main limitations of our study in the Abstract: “Our analyses did not include non-traditional vaccination sites and are based on data as of May 2021, thus they represent the early distribution of COVID-19 vaccines”.

5)	At this stage, we ask that you include a short, non-technical Author Summary of your research to make findings accessible to a wide audience that includes both scientists and non-scientists. The Author Summary should immediately follow the Abstract in your revised manuscript. This text is subject to editorial change and should be distinct from the scientific abstract. Please see our author guidelines for more information: [https://urldefense.com/v3/__https://journals.plos.org/plosmedicine/s/revising-your-manuscript*loc-author-summary__;Iw!!LLK065n_VXAQ!wMu1ryk3maZy-Y5cKkVkJfbV4UD9viL95JDt0q8ps4Vvv_W4KTS7eED8NbhryX_H9bi7$](https://urldefense.com/v3/__https:/journals.plos.org/plosmedicine/s/revising-your-manuscript*loc-author-summary__;Iw!!LLK065n_VXAQ!wMu1ryk3maZy-Y5cKkVkJfbV4UD9viL95JDt0q8ps4Vvv_W4KTS7eED8NbhryX_H9bi7$)

We have included an Author Summary after the abstract.

6)	Introduction: Please address and cite past research and explain the need for and potential importance of your study. Indicate whether your study is novel and how you determined that. If there has been a systematic review of the evidence related to your study (or you have conducted one), please refer to and reference that review and indicate whether it supports the need for your study.

We have summarized and cited prior research in the first two paragraphs of the introduction. We have also cited a review that synthesized the literature on determinants of equity in the uptake of COVID-19 vaccinations. We have explained the novelty of our research in the last paragraph of the introduction: “These past studies adopted a population focus in comparing the accessibility to COVID-19 vaccination sites across sociodemographic subgroups. Such approach is however limited in that it does not differentiate whether lower access in underserved neighborhoods is a product of the lower concentration of healthcare facilities in these areas or of inequities in the distribution of COVID-19 vaccines to facilities. To answer this question, we tested whether the likelihood of an eligible healthcare facility administering COVID-19 vaccines varied with the county-level racial/ethnic composition and degree of urbanicity”.

7)	Please conclude the “Introduction” with a clear description of the study question or hypothesis. The description of the database can be moved to the methods section

We changed the last paragraph of the introduction section accordingly: “We tested whether the likelihood of an eligible healthcare facility administering COVID-19 vaccines varied with the county-level racial/ethnic composition and degree of urbanicity. This approach enabled us to quantify equity in the distribution of COVID-19 vaccines to facilities. We hypothesized that healthcare facilities serving rural areas and underrepresented populations would be less likely to administer COVID-19 vaccines”.

8)	Did your study have a prospective protocol or analysis plan? Please state this (either way) early in the Methods section.

Our study did not have a prospective protocol, this has been stated in the last sentence of the first paragraph of the Methods section.

a) If a prospective analysis plan (from your funding proposal, IRB or other ethics committee submission, study protocol, or other planning document written before analyzing the data) was used in designing the study, please include the relevant prospectively written document with your revised manuscript as a Supporting Information file to be published alongside your study, and cite it in the Methods section. A legend for this file should be included at the end of your manuscript.

Not applicable.

b) If no such document exists, please make sure that the Methods section transparently describes when analyses were planned, and when/why any data-driven changes to analyses took place.

This information has been added to the methods section.

c) In either case, changes in the analysis-- including those made in response to peer review comments-- should be identified as such in the Methods section of the paper, with rationale.

We have documented in the Methods all changes made in the analysis in response to peer review comments.

9)	Please temper claims of primacy of results (e.g. “In the first nation-wide study…)” by stating, "to our knowledge" or something similar.

We have revised the language as directed: “To our knowledge, we present the first nationwide study to quantify disparities in the early distribution of COVID-19 vaccines to healthcare facilities”.

10)	Please ensure that the study is reported according to the STROBE and include the completed STROBE checklist as Supporting Information. Please add the following statement, or similar, to the Methods: "This study is reported as per the Strengthening the Reporting of Observational Studies in Epidemiology (STROBE) guideline (S1 Checklist)."

a)	When completing the checklist, please use section and paragraph numbers, rather than page numbers.

We have added the suggested statement in the methods section, and we have included the STROBE checklist as Supporting Information.

11)	How was race/ethnicity defined and by whom?

Our analyses included county-level measures of racial/ethnic composition, which were obtained the University of Wisconsin Population Health Institute County Rankings & Roadmap data. We have added this information to the methods section, Independent Variables subsection.

12)	You examined (as a binary outcome, yes/no) whether a facility serves as a covid-19 vaccination site; is there any data on the number of vaccines actually delivered?

We obtained the addresses of COVID-19 vaccine administration locations from the Centers for Disease Control and Prevention (CDC) VaccineFinder data. Unfortunately, these data did not include information on the number of vaccines delivered in each vaccination site. We have acknowledged this limitation in the discussion section: “CDC VaccineFinder did not have information on the number of vaccines delivered in each site. As a result, our outcome was defined as a binary variable (whether a facility served as a COVID-19 vaccine administration site), and we were not able to measure the number of vaccines delivered in each facility”.

13)	Do you have any insights into the reasons why particular sites might be signed up as vaccination sites or not

The data compiled from CDC VaccineFinder data only contained information on the addresses of COVID-19 administration sites. Therefore, it was not possible for us to determine the reasons why some of the health care facilities did not serve as vaccine administration sites. We have acknowledged this limitation in the discussion section: “The data compiled only contained information on the addresses of vaccine administration sites, so it was not possible to differentiate whether facilities did not serve as COVID-19 administration sites because of institutional or structural reasons”.

We have also discussed the need for future research to identify the reasons why facilitates did not serve as COVID-19 administration locations: “Identifying the drivers of the lower involvement of facilities serving underserved and rural areas in the administration of the COVID-19 vaccine is crucial to improve equity in the distribution of booster shots and of future public health prevention programs” (fifth paragraph of the discussion).

14)	Please discuss your analyses based on a hypothetical “Trump win” in 2020. For example, add a sentence to the effect that there were “no differences by voting preferences in the 2020 Presidential election”, or similar, assuming this is the case.

We have added a sentence as directed: “There were no differences in the likelihood of facilities serving as COVID-19 vaccine administration locations by voting results in the 2020 Presidential election (OR 0.96; 95% CI, 0.89-1.03, p=0.240 for counties with majority vote for Trump, compared to counties with majority vote for Biden).”

15)	Please provide p values in addition to 95% CIs in the main text and tables

We have added p-values in the text and Figure 1.

16)	Please indicate in the figure caption the meaning of the bars and whiskers in Figure 1.

This information has been added: “The circles represent the point estimate for the odds ratio, and the whiskers represent the 95% confidence interval”.

17)	Please present and organize the Discussion as follows: a short, clear summary of the article's findings; what the study adds to existing research and where and why the results may differ from previous research; include relevant citations; strengths and limitations of the study; implications and next steps for research, clinical practice, and/or public policy; one-paragraph conclusion.

We have organized the discussion as suggested.

18)	References:

a)	Please provide access dates for all references with a weblink

b)	Please select the PLOS Medicine reference style in your citation manager. In-text reference call outs should be presented as follows noting the absence of spaces within the square brackets,

c)	Please ensure that journal name abbreviations consistently match those found in the National Center for Biotechnology Information (NCBI) databases. [https://urldefense.com/v3/__https://journals.plos.org/plosmedicine/s/submission-guidelines*loc-references__;Iw!!LLK065n_VXAQ!wMu1ryk3maZy-Y5cKkVkJfbV4UD9viL95JDt0q8ps4Vvv_W4KTS7eED8NbhryfcIAqz1$](https://urldefense.com/v3/__https:/journals.plos.org/plosmedicine/s/submission-guidelines*loc-references__;Iw!!LLK065n_VXAQ!wMu1ryk3maZy-Y5cKkVkJfbV4UD9viL95JDt0q8ps4Vvv_W4KTS7eED8NbhryfcIAqz1$) .

We have formatted the references accordingly.

**Notes from the Academic Editor:**

The message and finding are important – that access is an issue.

The major limitation is the ecological analysis, and I would think important to ensure you have addressed things like Social Vulnerability Index (SVI). Adjustment for SVI may attenuate the race-access relationship, but I think it would not necessarily undermine the argument – it could be a mediator of the relationship with racialization.

We have now incorporated social vulnerability index in our analysis. The social vulnerability index includes 4 components: socioeconomic, household composition and disability, minority status and language, housing type and transportation. We have incorporated indicator variables for counties in the top quintile of the socioeconomic component, counties in the top quintile of the household composition and disability component, and counties in the top quintile of the housing type and transportation component. We did not include a variable for the minority status and language component of the social vulnerability index because minority status is already captured by the measures of racial/ethnic composition defined above. The incorporation of these variables is described in the methods section. The results are shown in Figure 1.

**Comments from the reviewers:**

**Reviewer #1:** The authors have applied geographic information systems methods to test whether the likelihood of an eligible healthcare facility administering COVID-19 vaccines varied with the county-level racial composition and degree of urbanicity.

Comments:

"The outcome was regressed against county-level measures for racial/ethnic composition, urbanicity, income, COVID-19 mortality, 2020 election results, and availability of non-traditional vaccination locations using generalized estimating equations."

Did the authors consider including covariates in the models in order to adjust for potential confounding, such as county- or facility- level age distribution, comorbidity prevalence, and gender ratio?

Following the editor’s comment, we have included indicator variables for counties in the top quintile of the socioeconomic, household composition and disability, and housing type and transportation components of the social vulnerability index. County age distribution and proportion of residents with disability are included in the calculation of the household composition and disability component of the social vulnerability index. We did not add any additional variables because we believe the suggested domains are accounted for by our incorporation of social vulnerability index components. Nevertheless, in response to a comment from reviewer #3, we conducted sensitivity analyses where we added an indicator variable for counties in the top quintile of proportion of population older than 65 years of age (please see table in page 13 of this response letter).

"The outcome -whether an eligible vaccination facility was registered as actually administering COVID19 vaccines—was regressed against covariates listed above using logistic regression. We applied generalized estimating equations to account for clustering of facilities within a county."

The authors have applied a technically appropriate modelling technique for the data and research question in hand.

Thank you.

"We tested interactions between racial/ethnic composition and urbanicity. Because some FQHCs, RHCs, and HODs only offered COVID-19 vaccines to registered patients, we constructed one set of analyses for all four types of healthcare facilities and a second set for pharmacies only."

The authors have undertaken several additional analyses that help to demonstrate the robustness of the study findings.

Thank you.

Can the authors please present statistical findings (i.e. exact p-values and CIs) for all stated study inferences throughout the Results text?

We have now added exact p-values to the text the Figures.

Did the authors consider exploring 'County-Level Proportion of Black Population' and 'County with Proportion Hispanic Population' as continuous variables within the analysis?

We categorized these variables because: 1) they are not normally distributed (see histograms below); 2) we believe that the effect of these variables in the outcome is not linear, as demonstrated by the differences in estimates observed across categories; 3) our results for the interaction between urbanicity and proportion of Black or Hispanic population would be more difficult to interpret.

Reviewer #2: I enjoyed reading this manuscript and I believe it is worthy of acceptance and publication in PLOS Medicine. I liked how the authors side-stepped the limitations regarding what data the federal government has regarding reported vaccination administrations with regards to race and ethnicity. However, before publication, I believe the manuscript can be strengthened. Most, if not all, of these suggestions should not require reanalysis, but may require some reorganization and a few more sentences. I think there is enough to call it a major revision, but hopefully not one that is a heavy lift:

The authors state their goal is to measure health equity in the "actual distribution of COVID-19 vaccines." If that is the ultimate goal, then the portion that is pharmacies or alternate facilities is less important than the overall totality of vaccination sites and how those impact minorities. If the goal is also to highlight that, after COVID-19 ends, there may be pullback and these variations in how vaccines are administered will be more important for 'normal' vaccinations, this could be stated more clearly. I suggest this is a secondary focus because of a sentence in the discussion that states the objective was to measure the likelihood of existing healthcare facilities providing vaccinations. This objective is different from the "actual distribution of vaccines" and makes the later discussion of improved geographic access to vaccination fall somewhat flat. For example, yes, there is a continuum for pharmacies for the Black population (Figure 1a), but for whatever reason this only holds significantly for the greater than 42.7% group for all health care facilities (and here that confidence interval is a 'squeaker.') The same issue for pharmacies versus all four types of facilities does not seem to exist for either the Rural or the COVID mortality. Pragmatically, if the only goal is to get COVID vaccines into arms, then who is delivering those vaccines does not matter. If you want to draw conclusions about what it looked like at the beginning of the pandemic, what the current coverage says about how the system is responding to inequities (and here you'd need to spell out why a pharmacy vaccination has a different connotation than a federal facility, etc.), or how vaccinations will look after, then who is giving the vaccines matters more.

The goal of the paper is to measure equity in the distribution of COVID-19 vaccines, not to speculate about administration of vaccines beyond the COVID-19 pandemic. We have specified the objective of our analysis in the last paragraph of the introduction section: “we tested whether the likelihood of an eligible healthcare facility administering COVID-19 vaccines varied with the county-level racial composition and degree of urbanity”.

We agree with the reviewer that the important goal is to achieve equity in the distribution of COVID-19 vaccines, regardless of facility type. However, we believe that performing and reporting subgroup analyses for community pharmacies is important because the remaining facility types (federally qualified health centers, rural health clinics and hospital outpatient departments) in some cases only offered vaccines to registered patients. We have noted this in the methods section: “Because some FQHCs, RHCs, and HODs only offered COVID-19 vaccines to registered patients, we constructed one set of analyses for all four types of healthcare facilities and a second set for pharmacies only”. Nevertheless, we have dropped from the discussion section the comment on the gradient effect observed for the likelihood of pharmacies serving as vaccine administration sites by the proportion of Black population in metropolitan areas.

Continuing on this theme, the authors do acknowledge non-traditional vaccine distribution sites and availability of other vaccine distribution locations, but as a reader I don't think I fully understood the implications in the text. Here, I'm trying to highlight reader confusion, rather than give exact instructions on how to address that confusion. What do these variable these mean along the rural to urban spectrum. What does 500m mean in a rural location? Is there some kind of relationship for rural locations having non-traditional sites? This is not primary to the manuscript, but I didn't feel comfortable that I understood what these variables really meant. They appear again in Figure 1A. Since I don't know what they mean, when I read their odds ratios, I am guessing that having them in a county means more people are getting vaccinated than one would expect? Does this imply they are likely geographic overcompensation? Does the philosophy of vaccine distribution sites differ for low density rural areas, where one would expect long drives to pretty much everywhere? Or does it not differ because you can't expect everyone to have cars?

We apologize for the confusion. Our sample did not include non-traditional vaccination sites such as stadiums or conference centers; however, we adjusted for the density of alternative vaccination locations. In other words, we accounted for the fact that healthcare facilities in counties heavily relying on non-traditional settings may have been less likely to administer COVID-19 vaccines. We have explained this in the methods section: “Healthcare facilities in counties relying on non-traditional settings for vaccination such as stadiums or convention centers may have been less likely to administer vaccines. To account for this, we adjusted analyses for the density of alternative vaccination locations”.

We also explained the meaning of the variable that captures the presence of COVID vaccine administration facility within 500m: “Finally, a healthcare facility may have been less likely to administer COVID-19 vaccines if there was another COVID-19 vaccination location nearby. Thus we adjusted for the availability of a COVID vaccine administration facility within 500m of a given facility”.

Another issue that should be addressed, if not accounted for possibly, is that CDC and Census report "Black" as a race and "Hispanic" as an ethnicity. This means that Hispanic includes Hispanic Blacks and Black includes Hispanic Blacks. I am guessing that the authors ignored this nuance because this group of people is not large compared to the total for either Black or Hispanic. Or because, for example, Florida doesn't report its data like Census, etc. This Race/Ethnicity detail should probably be acknowledged before being ignored (caveat: this is a pet peeve)

We apologize for the omission, the proportion of Black population actually represents non-Hispanic Black population. We have made this change throughout the manuscript.

The results, conclusions, and Figure 1 should also focus on Hispanics, as they are mentioned in the text. There should be a paragraph in results about the Hispanics that mirrors that for Blacks, at least a sentence in the conclusions, and probably a 1C section for Hispanic data that mirrors 1B. My assumption is that the data showed no significantly different odds ratios for Hispanics, so all of this was stricken for not showing a relationship. If so, the data cannot reject lack of a health equity issue (null hypothesis). If one goes looking for a health equity issue for Hispanics and there isn't a visible relationship that supports one's assumptions for Hispanics, that should probably be explicitly stated or there is a risk of appearing to "cherry pick" to meet expectations coming into the scholarship. It might also be worth noting why other groups were not considered (e.g. Non-Hispanic Asians, which might flip the other direction). I'm assuming it was an issue of sample size, rather than an a priori assumption that only Blacks and Hispanics would be significant.

We added a similar Figure for the interaction between Proportion of Hispanic Population and urbanicity, as requested by the reviewer (Figure 3). We have described the results in the last paragraph of the results section: “We also identified a significant interaction of urbanicity with Hispanic composition: In rural counties with above average Hispanic composition, healthcare facilities had 26% (95% CI, 11%-38%, p=0.002) lower odds of serving as COVID-19 vaccine administration locations compared to facilities in counties with below average Hispanic composition (Figure 3). However, this disparity was not observed in metropolitan or suburban counties.”

We have noted as a limitation of our analyses our lack of adjustment for the proportion of racial/ethnic subgroups other than Non-Hispanic Black and Hispanic. The reviewer is correct, these were not included due to the low proportion of other racial/ethnic groups in most US counties: “Our evaluation of racial/ethnic disparities focused on Non-Hispanic Black and Hispanic due to the low proportion of other minority groups in most counties, which yielded unstable estimates.”

This dovetails into the bigger point regarding the Discussion. The implications of Figure 1B should guide more of the conclusions of the manuscript. I think the existing first two sentences at the start of the discussion are fine. But it should be spelled out that, when combining Black composition and urbanicity, only >42.5 was significant.

We have revised the summary of results in the first paragraph of the discussion section as follows: “We observed however significant interactions between urbanicity and demographic composition: The county-level proportion of Black population was associated with decreased odds of a facility administering COVID-19 vaccines in metropolitan but not in suburban or rural counties. The county-level proportion of Hispanic population was associated with decreased odds of a facility administering COVID-19 vaccines in rural but not in metropolitan or suburban counties.”

We have not specified that only the >42.2% Black composition group is significant as suggested by the reviewer because the 12.5%-42.2% group is also significant for the model constructed for community pharmacies.

The existing sentence points out there is a continuum for metropolitan areas, but it does not indicate that neither Suburban nor Rural were significantly different for Blacks. Nor does it note, apparently, that the authors could not find such a relationship for Hispanics. This should be noted, but the authors then have the opportunity to explain why a relationship was not found (brainstorming rather than statements of fact: data limitations, underreporting of Hispanics in Rural areas that use migrant workers, Blacks really are the special case here and being Hispanic/Latino doesn't matter, there's a difference between being Hispanic in some counties rather than others—kinds of work, illegal vs. citizen, etc.—that is obscuring a real relationship).

We have acknowledged in the first paragraph of the discussion section that the disparity by Black race was not observed in suburban or rural counties and that the disparity by Hispanic ethnicity was not observed in metropolitan or suburban counties: “The county-level proportion of Black population was associated with decreased odds of a facility administering COVID-19 vaccines in metropolitan but not in suburban or rural counties. The county-level proportion of Hispanic population was associated with decreased odds of a facility administering COVID-19 vaccines in rural but not in metropolitan or suburban counties”.

The significant mortality odds ratio is almost self-explanatory (less people vaccinated means more die), but this is not explicitly stated. Nor is the meaning of the non-traditional vaccine distribution sites and availability of other vaccine distribution locations. Are non-traditional sites appearing in counties that otherwise would be 'vaccine deserts' and therefore indicate that decision makers realized they had a problem in these locations and shored up vaccine coverage? Does the less than 500m calculation matter at all in rural locations? All of this also feeds into your abstract conclusion, which could be more specific.

The mortality data was obtained as May 10, 2021. Therefore the indicator variable for counties in the top quintile of COVID-19 mortality represents hardest-hit communities prior to the date of study. We have clarified this point in the methods section: “We created an indicator variable for counties in the top quintile of COVID-19 mortality, which represent communities that were hardest-hit by COVID-19 prior to the date of our analysis.”

We explain the meaning of the variables capturing other distribution sites in the methods section: “Healthcare facilities in counties relying on non-traditional settings for vaccination such as stadiums or convention centers may have been less likely to administer vaccines. To account for this, we adjusted analyses for the density of alternative vaccination locations. Alternative vaccine administration locations were defined as vaccination locations reported by CDC in VaccineFinder data that did not match to pharmacies, HODs, RHCs, or FQHCs. Finally, a healthcare facility may have been less likely to administer COVID-19 vaccines if there was another COVID-19 vaccination location nearby. Thus we adjusted for the availability of a COVID vaccine administration facility within 500m of a given facility”.

We did not fit interactions between the availability of a COVID vaccine administration facility within 500m and urbanicity because our primary analysis already includes interactions between urbanicity and racial/ethnic composition and the fitting of three way interactions would yield complex results to interpret.

Less pressing suggestions:

Please include line numbers on the next draft. I can't give you specific feedback about typos, etc, because it is hard to direct you.

We have now included line numbers.

Please provide the reader with a stage setting of what kinds of vaccine sites are seen nationally and then dive into the data you have for those sites. For example, what about pop-up clinics, what about vaccines at long term care facilities? Indicate to me that you have considered/know about all of the options and how they fall into your categories.

We added the number of facilities by type in the first paragraph of the results section: “The sample included 50,806 community pharmacies, 11,619 FQHCs, 3,187 HOPDs, and 1,255 RHCs distributed across 2,942 counties in US states”.

The objective of our analysis is to test whether the likelihood of open-door healthcare facilities administering COVID-19 vaccines differed with the sociodemographic composition of the population served. Therefore, we did not include vaccination sites other than community pharmacies, hospital outpatient departments, rural health clinics, and federally qualified health centers in our analysis. We acknowledged this limitation in the discussion section: “Our sample did not include non-traditional vaccination sites because our objective was to measure the likelihood of existing healthcare facilities serving as COVID-19 vaccine administration locations”.

Pop-up clinics are not existing health care facilities and therefore were not included in analyses. Long term facilities are not open door facilities where community dwelling individuals can be vaccinated and therefore were not included in our sample. We have now stated this in the first paragraph of the methods section: “Long-term facilities were not included because they are not available for the vaccination of community dwelling individuals”.

PLOS Medicine may want outcomes first in the abstract and methods (I am guessing), but if not, it would be better to set the stage before going straight to them.

We have not rearranged the methods or abstract as this has not been noted in the formatting edits requested by the editor. However, we would be happy to do so if requested by the editor.

Was it really defined as "indicator of Trump Win?"

Based on election results, we created an indicator variable representing whether Trump won each county. We have clarified this in the methods section: “We created an indicator variable for counties where Trump (Republican candidate) won the 2020 presidential election”. We have also renamed this variable in Figure 1 to avoid confusion.

The second paragraph of the results could be more explicit that it is discussing 'all 4 facility types', if that is what it is doing.

We have not made the requested change because if we state “In adjusted analyses, the four types of health care facilities examined were less likely to serve as COVID-19 vaccine administration locations” readers may interpret that we conducted four sets of analyses, one for each facility type, which we did not.

You might want to give more basic numbers (ideas rather than exact guidance follows): How many counties were you looking at? if there are some you had to remove, state that or throw up a map showing your data gaps to assure the reader that you are being close to comprehensive. Are you just looking at the US States? What about USVI, Puerto Rico, American Samoa, etc.? How many Blacks, how many Hispanics?

There were no individual level data included in analyses, therefore we cannot report individual level data by race and ethnicity. We have provided summary statistics in the first paragraph of the results section regarding facilities and we have stated that US territories were not included: “The sample included 50,806 community pharmacies, 11,619 FQHCs, 3,187 HOPDs, and 1,255 RHCs distributed across 2,942 counties in the US (only states, territories were not included).”

Other potential data issues that might be worth addressing: I can't think of exactly how it would affect your data overall, but people jump counties for vaccines (go into a city for work, the clinic is one county over, but close to home, etc.). Also, people jump channels (pharmacy to federal facility, etc.). Maybe this noise is self-limiting in your data, I don't know if this is worth addressing or not. If you have taken data from the beginning until now, do you need to account for individuals getting up to three doses at this point or successively wider age ranges for vaccination? Is there some kind of significant relationship for the J and J one shot vaccine going into a particular population or urbanicity that skews your results?

Our analyses are not conducted at the person level but at the facility level. We agree with the reviewer that individuals can cross county lines to access vaccine sites; however, this is not a major concern of our study because we are not measuring access at the person level (we are not measuring distance from an individual’s home address to the nearest COVID-19 administration site). Instead, we are testing whether the likelihood of a healthcare facility administering COVID-19 vaccines differed with the sociodemographics of the population served.

Our outcome is defined as whether a facility served as vaccine administration location, we were not able to identify the specific vaccine types offered at each facility. We have acknowledged this limitation in the discussion section: “We did not evaluate variation in the types of vaccines distributed to each health care facility”.

This is probably beyond your scope, but it would be interesting in a figure showing vaccines over time for Blacks and Hispanics. As COVID-19 has gone on, has the country done a better job at addressing inequities at the county level? Is there a way to display that?

Our study is a cross-sectional analysis that captured the early experience in the distribution of COVID-19 vaccines to existing health care facilities, we have acknowledged this limitation repeatedly throughout the text (please see our response to the editor’s comment in page 1).

**Reviewer #3:**

The authors present a study of COVID-19 vaccination sites, with the goal of evaluating fairness of vaccine access.

Major comments

Introduction

Please include a summary of or at least references to one of the many frameworks that were created prior to COVID-19 vaccine availability to support equitable distribution of vaccines, e.g., PMID: 3237895. In the discussion section, consider commenting upon our success at following the goals laid out in one or more of these frameworks. Additionally, it's important to recall that the US allowed COVID-19 vaccine eligibility in phases; this should be mentioned in the introduction as it likely affected placement of vaccine sites.

The PMID provided does not correspond to a paper on equity in the distribution of COVID-19 vaccines (the title of the study with PMID 3237895 is “Psychosocial management of deaf patients”). Nevertheless, we have now cited and summarized in the introduction the framework for the equitable allocation of COVID-19 vaccines by the National Academy of Science, Engineering and Medicine: “The distribution of COVID-19 vaccines in the US followed the framework proposed by the National Academies of Science, Engineering, and Medicine, which prioritized in early phases of vaccine distribution health care workers and long-term care residents, followed by older adults, frontline workers, those with high-risk conditions, and essential workers. For each population group, the framework called for a prioritization of vulnerable areas which have been disproportionately impacted by the COVID-19 pandemic”.

The authors state, "to our knowledge, no nationwide studies have measured health equity in the actual distribution of COVID-19 vaccines to healthcare facilities;" however, studies taking a different approach to evaluation of health equity in vaccine distribution have been published and are easily accessible, e.g., PMID: 34213561. The authors should consider a more thorough literature review to provide the reader with a succinct summary of the work that has already been done on health equity and vaccine distribution in the US.

We have now performed a more thorough literature search and included a description of prior work in the first two paragraphs of the introduction. We have included the citation provided by the reviewer: “Although the US public generally agreed with the prioritization of disadvantaged communities hardest hit by COVID in vaccination, most COVID-19 distribution plans were created without input from health equity experts”.

Methods and Results

As this study only evaluated vaccination sites in place as of May 2021, it's important to consider how eligibility for and availability of vaccines changed over time ([https://urldefense.com/v3/__https://www.cdc.gov/vaccines/imz-managers/downloads/Covid-19-Vaccination-Program-Interim_Playbook.pdf__;!!LLK065n_VXAQ!wMu1ryk3maZy-Y5cKkVkJfbV4UD9viL95JDt0q8ps4Vvv_W4KTS7eED8NbhryaDEdoV7$](https://urldefense.com/v3/__https:/www.cdc.gov/vaccines/imz-managers/downloads/Covid-19-Vaccination-Program-Interim_Playbook.pdf__;!!LLK065n_VXAQ!wMu1ryk3maZy-Y5cKkVkJfbV4UD9viL95JDt0q8ps4Vvv_W4KTS7eED8NbhryaDEdoV7$) , see page 12), and to note that this varied by county. If possible, it would be helpful for the authors to compare their data to similar data later in 2021 or in early 2022. If this is not possible, the study should be clearly characterized as an early evaluation of vaccine sites, e.g., in the first sentence of the Results section should read, "…provided COVID-19 vaccinations as of May 2021."

Unfortunately, we are not able to update our results with more recent data. However, we believe our data is of main relevance because it represents the distribution of COVID-19 vaccines in the first wave of vaccination, when the general public became eligible for vaccination. We have acknowledged that our findings represent the early distribution of COVID-19 vaccines repeatedly throughout the text (please see our response to editor’s comment in page 1). We have also stated in the discussion section that future studies are necessary to test whether disparities have persisted over time.

Consider showing the outcome(s)/independent variables stratified by the population types that were eligible for vaccination during their study period, which ended in May 2021 (therefore, only 3-4 months after COVID-19 vaccines were available to only certain portions of the population [e.g., healthcare workers who likely were vaccinated at their places of employment, and the elderly, who were likely vaccinated at their physician's office). In other words, during the time when vaccines were only available for healthcare workers and people >65, were vaccines easily accessible to the corresponding elderly portion of the population?

Our study is of a cross sectional nature and represents the early distribution of COVID-19 vaccines. All adults were available for vaccination in May 2021.[1] We did not stratify variables by population types because our sample does not include individuals but facilities.

We conducted sensitivity analyses where we added an indicator variable for counties in the top quintile of proportion of population older than 65 years of age. This variable was not significant, which demonstrates that healthcare facilities serving older populations were not more likely to serve as vaccine administration sites (please see table below). We have not included this indicator variable in the main analyses because the proportion of county residents>65 years is included in the calculation of the household composition and disability component of the social vulnerability index.

Adjusted Odds Ratio for Healthcare Facilities Serving as COVID-19 Vaccine Administration Locations.

|  | Odds Ratio (95% CI) | p-value |
| --- | --- | --- |
| County-Level Proportion of Black Population |  |  |
| <12.5% | Ref |  |
| 12.5%-42.2% | 1.03 (0.96-1.11) | 0.354 |
| >42.2% | 0.83 (0.70-0.98) | 0.029 |
| County-Level Proportion of Hispanic Population |  |  |
| <18.5% | Ref |  |
| 18.5%-38.7% | 0.93 (0.86-1.01) | 0.077 |
| >38.7% | 0.90 (0.78-1.05) | 0.182 |
| Urbanicity |  |  |
| Metropolitan | Ref |  |
| Suburban | 1.02 (0.95-1.10) | 0.605 |
| Rural | 0.83 (0.76-0.91) | <0.001 |
| Facility in County in Top Quintile for Proportion Population >65 years | 0.95 (0.86-1.04) | 0.242 |
| Facility in County in Bottom Quintile for Median Income | 0.89 (0.78-1.01) | 0.072 |
| Facility in County in Top Quintile for Vulnerability Index - Socioeconomic Component | 1.06 (0.93-1.21) | 0.415 |
| Facility in County in Top Quintile for Vulnerability Index - Household Composition & Disability | 1.01 (0.91-1.12) | 0.836 |
| Facility in County in Top Quintile for Vulnerability Index -Housing Type & Transportation | 0.95 (.88-1.02) | 0.156 |
| Facility in County in Top Quintile for COVID Mortality | 0.83 (0.75-0.93) | 0.001 |
| Facility in County with 2020 Trump Win | 0.96 (0.90-1.03) | 0.262 |
| Non-traditional Vaccine Distribution Locations per 10,000 | 1.43 (1.35-1.52) | <0.001 |
| Availability of Vaccine Distribution Location within 500m | 1.23 (1.17-1.29) | <0.001 |

Additionally, it would be helpful for the authors to comment upon the dates of their independent variable data. E.g., did they obtain the COVID-19 mortality data from the Johns Hopkins dashboard in or before May 2021 so that it temporally corresponded with their vaccine site data?

COVID-19 mortality data was obtained as of May 10, 2021, the same day as the CDC VaccineFinder data. This has now been noted in the methods: “COVID-19 mortality was obtained as of May 10, 2021 from the John Hopkins University Coronavirus Resource Center”.

The table suggests that proportions of vaccine sites available to counties with varying levels of Black/Hispanic population did not vary significantly (e.g., for counties with black population <12.5%, 12.5%-42.2%, and >42.2%, the percent of healthcare facilities that served as vaccine sites was 60.5%, 63.9%, and 55.5%, respectively. It would be helpful for the authors to include a statistical comparison of the differences in these proportions and comment upon any significant findings, rather than merely mentioning the proportions of select groups in the Results section.

The table only shows summary statistics, the results of adjusted statistical comparisons are shown in

Figures 1-3. We have removed from the text the mentions of these unadjusted proportions, and we have focused on describing the results of adjusted analyses.

Discussion

Again, the authors' results should be characterized as "early" if they are unable to show later analyses for comparison. E.g., in the first sentence of the Discussion, they should write "...disparities in the early distribution…"

We have made the requested change: “To our knowledge, we present the first nationwide study to quantify disparities in the early distribution of COVID-19 vaccines to healthcare facilities”.

Consider offering suggestions as to why the findings may be true. E.g., what barriers challenged availability of COVID-19 vaccination centers in the "hardest hit" communities? Were early COVID-19 vaccination sites targeted at locations with concentrated populations of people >65yo regardless of race because only people >65yo were eligible for vaccination at the time of the study?

Unfortunately, the data compiled did not have information on reasons why facilities did not serve as COVID-19 vaccine administration sites. We have acknowledged this limitation in the discussion section: “The data compiled only contained information on the addresses of vaccine administration sites, so it was not possible to differentiate whether facilities did not serve as COVID-19 administration sites because of institutional or structural reasons”. We have also discussed the need for future research to identify the reasons why facilitates did not serve as COVID-19 administration locations: “Identifying the drivers of the lower involvement of facilities serving underserved and rural areas in the administration of the COVID-19 vaccine is crucial to improve equity in the distribution of booster shots and of future public health prevention program” (fifth paragraph of the discussion).

Please see in page 13 the results of sensitivity analyses where we added an indicator variable for counties in the top quintile of proportion of population older than 65 years of age. This variable was not significant. We have not included this variable in the main analyses because the proportion of county residents>65 years is included in the calculation of the household composition and disability component of the social vulnerability index.

It doesn't make sense to evaluate availability/accessibility of vaccine sites when most of the county population was not eligible for vaccination. For most of the study period, which ended in May 2021, only a relatively small proportion of the population was eligible in most counties (e.g., healthcare workers, the elderly, people with certain medical conditions). I think it's important that the authors discuss (and hopefully address by updating their data/analysis!) these limitations in the discussion section. In the last paragraph of the Discussion they provide a call to action that may not be necessary if vaccine equity did in fact improve after the study period. Ideally the authors would apply the same methods to evaluate health equity at vaccine sites at a later period as well, e.g., in late 2021 and/or early 2022.

All US adults were eligible for COVID-19 vaccination on or before May 1, 2021, the deadline set by President Biden. We have acknowledged throughout our manuscript that our results represent the early distribution of COVID-19 vaccines (please see our response to the editor’s comment in page 1).

Reviewer #4: OVERALL COMMENTS:

This is an important and well-written study that examined the equity in vaccine districubtion across health care facilities and pharmacies in the US. The study examined equity in the distribution of COVID-19 vaccines across healthcare facilities and pharmacies in the US. They found the distribution of vaccines differed by geographic and racial composition.

INTRODUCTION:

The introduction is too brief and does not fully expand on the scope and significance of the problem. Even a descriptive report on who received COVID-19 vaccines to date would be helpful. There had been a recent study on public opinion of vaccine distribution prioritization in 2021 that may be useful to cite.

Would also be helpful to state why measuring health care facilities is important beyond who received the vaccines. You state this later in the manuscript, but this is important to describe in the introduction.

In addition, the paper is focused on the health facilities that were registered to distribute. It may also be helpful to briefly mention the barriers to the health care facilities getting registered and how this may exacerbate disparities?

We have expanded the introduction section to describe the significance of the problem and we have summarized prior research. We have cited the study of public opinion of vaccine prioritization suggested by the reviewer: “Although the US public generally agreed with the prioritization of disadvantaged communities hardest hit by COVID in vaccination, most COVID-19 distribution plans were created without input from health equity experts.”

We have explained why our facility level analysis is important in the last paragraph of the introduction section: “These past studies adopted a population focus in comparing the accessibility to COVID-19 vaccination sites across sociodemographic subgroups. Such approach is however limited in that it does not differentiate whether lower access in underserved neighborhoods is a product of the lower concentration of healthcare facilities in these areas or of inequities in the distribution of COVID-19 vaccines to facilities. To answer this question, we tested whether the likelihood of an eligible healthcare facility administering COVID-19 vaccines varied with the county-level racial/ethnic composition and degree of urbanicity.”

METHODS:

Under data sources:

Please operationalize vaccine distribution equity, this will help ground the results.

Our study focused on assessing vaccine distribution inequity, which we measured as differences in the likelihood of an eligible healthcare facility administering COVID-19 vaccines by the sociodemographic composition of the population served. We have stated the relevant information in the last paragraph of the introduction section.

Please define the acronyms for HOD and RHC.

Both are defined in the first paragraph of the methods section. HOPD stands for hospital outpatient departments and RHC for rural health clinics.

The authors indicate the study was exempt, was this determined by and IRB? If so, please state that.

No human data were used in analyses. Data included facility addresses and county level characteristics; therefore, the study did not constitute human subjects research. The study was not submitted to IRB because IRB review is only required by federal regulation for projects that meet the definition of human subjects research.

Under independent variables:

It is not clear why election results were included. A sentence or two to explain this was included.

Election results were included as an indicator variable to capture the majority political affiliation of the area served by a facility. This has been now specified in the methods: “We created an indicator variable for counties where Trump (Republican candidate) won the 2020 presidential election. This variable was included in analysis to capture the majority political affiliation of the area served by a facility”.

Can you provide an example of an alternative vaccine distribution location? The authors define what they were not (HOD, RHC and FQHCS), but not what they were.

This is provided in the methods section, independent variables subsection: “Healthcare facilities in counties relying on non-traditional settings for vaccination such as stadiums or convention centers may have been less likely to administer vaccines”.

RESULTS:

In the figure, a key for the shapes (triangle, circle, square) would be helpful.

This has been added.

DISCUSSION:

In the first sentence of the Discussion section. I believe it should read "…quantify disparities in the distribution locations of COVID-19 vaccines, …".

We have rephrased this sentence following all reviewers’ comments.

The interpretation of the interaction terms should be mentioned.

This has been added: “We observed however significant interactions between urbanicity and demographic composition: The county-level proportion of Black population was associated with decreased odds of a facility administering COVID-19 vaccines in metropolitan but not in suburban or rural counties. The county-level proportion of Hispanic population was associated with decreased odds of a facility administering COVID-19 vaccines in rural but not in metropolitan or suburban counties.”

The data is limited to the first quarter of vaccine distribution to the general public (May 10, 2021). This should be discussed in the limitations. Distribution may have improved over time.

We have acknowledged throughout the manuscript that our results capture the early distribution of COVID-19 vaccines and noted this as a limitation of our study. We have also stated that future studies are necessary to test whether disparities persisted over time (please see our response to the editor’s comment in page 1).

Also worth mentioning the role of preference for where to access, resources in rural and safety-net locations. A location to distribute and resources to distribute are both important.

We acknowledge that the preference for where to access and the availability of resources play important roles in vaccine access. Our study cannot accommodate these analyses because our data are at the facility level. We have addressed this limitation in the discussion: “our data and analyses were at the facility level rather than at the person level so we were not able to assess individual preferences in accessing healthcare facilities for vaccination”.

The odds ratios are helpful but not sufficient. It is not clear what the impact is. Ground the results in how many lives are impacted by the odds.

Our analyses were conducted at the facility level, not at the person level. Due to the unavailability of data on the number of individuals served by each facility, it is not possible to calculate the number of people impacted.

**Reviewer #5:** Overall, this article poses an interesting research question but lacks details and justification about their Methods which would be used to judge their Results. Specific comments include:

The Introduction is very sparse on why the variables they chose to investigate are important and relevant. This should be built out more.

We have expanded the introduction following the reviewers’ and editor’s comments.

This sentence need a citation: "County-level measures of racial/ethnic composition included proportion Black and proportion Hispanic, and were categorized in three levels following CDC methodology:(6) 1) below US average; 2) between US average and 95th county percentile; 3) above 95th county percentile."

The citation has been added, we apologize for the oversight.

Authors do not include their variable selection methodology for the final logistic regression model.

We did not use any variable selection method. We have clarified this in the methods section: “All independent variables specified above were included in the final model”.

Authors do not include counts of facilities, etc. in the Results section write-up. This is important for readers to understand how big these sample sizes are and to determine if the Methods are sound.

We have added counts of facilities in the first paragraph of the results section.

There are no p-values in the text - unless excluding them is part of the journal style, this is essential to include.

We have added p-values.

What proportion of the U.S population falls into metropolitan vs. suburban vs. rural counties? This is an important comparator.

This has now been added to the methods section: “Urbanicity was categorized in 3 levels using US Department of Agriculture Rural-Urban Continuum Codes (RUCC), as previously done by CDC: 1) metropolitan (RUCC code 1, accounts for 168.5million people); 2) suburban (RUCC codes 2-3, accounts for 93.9million people); 3) rural (RUCC codes 4-9, accounts for 46.3 million people)”.

**Reviewer #6:** General impression: This is a brief report of findings on variability in distribution of vaccinates from eligible to distribution centers/pharmacies. The written presentation is clear and presents the data well. The data presentation is difficult to interpret without the scale (how many pharmacies and facilities were examined and considered eligible). The ability to post the publicly available data on the number of eligible pharmacies and their distribution would strengthen the ability to replicate the findings.

As noted in the data statement, data from community pharmacies were obtained from the National Council for Prescription Drug Programs under a license. Researchers can access the data from National Council for Prescription Drug Programs after purchasing a license. However, remaining data sources are publicly available, and the links have been provided in the data statement.

Abstract: The abstract is written clearly and coveys the key findings well. One point that is not clear is the interpretation of the significant interaction between urbanicity and Black area-level composition - the interpretation of that interaction term should be described more clearly.

We have rephrased this point to improve clarity: “There was a significant interaction of urbanicity and racial/ethnic composition: In metropolitan counties, facilities in counties with >42.2% Non-Hispanic Black population (i.e., > 95th county percentile of Black race composition) had 32% (95% CI 14%-47%, p=0.001) lower odds of serving as COVID-administration facility compared to facilities in counties with below US average Black population. This association between Black composition and odds of a facility serving as vaccine administration facility was not observed in rural or suburban counties.”

Objectives: The study objectives are to understand the "likelihood of an eligible healthcare facility administering COVID-19 vaccines varied with the county-level racial composition and degree of urbanicity." However, the introduction reads that the study motivation is to better understand the "actual distribution of COVID-19 vaccines to healthcare facilities." These are related but different concepts with different policy implications. What led to the distribution of vaccines (structural problem)? Why did specific institutions or facilities administer vaccines (institutional or structural issue)? The objectives in the introduction might be helped by additional information on the hypothesis, and any evidence supporting the interpretation of the hypothesis.

We have rewritten the introduction section to frame the topic more clearly and to explain why facility level analyses are relevant: “These past studies adopted a population focus in comparing the accessibility to COVID-19 vaccination sites across sociodemographic subgroups. Such approach is however limited in that it does not differentiate whether lower access in underserved neighborhoods is a product of the lower concentration of healthcare facilities in these areas or of inequities in the distribution of COVID-19 vaccines to facilities. To answer this question, we tested whether the likelihood of an eligible healthcare facility administering COVID-19 vaccines varied with the county-level racial/ethnic composition and degree of urbanicity.”

We have added our hypothesis to the last paragraph of the introduction section: “We hypothesized that healthcare facilities serving rural areas and underrepresented populations would be less likely to administer COVID-19 vaccines.”

We have acknowledged in the discussion section that we are not able to differentiate whether facilities did not serve as COVID-19 administration sites because of institutional or structural reasons. We agree with the reviewer that this is an important issue and so we have stated in the discussion the need for future research to identify the reasons why facilitates did not serve as COVID-19 administration locations: “Identifying the drivers of the lower involvement of facilities serving underserved and rural areas in the administration of the COVID-19 vaccine is crucial to improve equity in the distribution of booster shots and of future public health prevention program”.

Methods:

The methods do not describe how vaccination sites that were not listed on VaccineFinder were found. If these clinics were not included, this should be noted as a data limitation in the discussion section.

We have acknowledged this limitation in the discussion section: “Several limitations of our study are related to the data sources, and particularly to the use of CDC VaccineFinder data for the identification identify actual vaccine administration locations. First, locations not registered in VaccineFinder were not identified, which could have led to an underestimation of the proportion of healthcare facilities serving as vaccine administration locations.”

The language for some of the concepts should be reviewed and clarified for an international audience (what is a Trump win? Is that to indicate Republican, Democratic or Independent candidate for elected office/president?)

We have now clarified the definition of this variable: “Results of the 2020 election were obtained from the MIT Election Data and Science Lab.[26] We created an indicator variable for counties where Trump (Republican candidate) won the 2020 presidential election. This variable was included in analysis to capture the majority political affiliation of the area served by a facility”.

The limitation section should discuss some of the assumptions made calculating non-pharmacy locations (for example, Gillette Stadium, a large football field, was used to distribute vaccines in Massachusetts).

We have acknowledged in the discussion section that non-traditional vaccine sites were not included in the sample: “Our sample did not include non-traditional vaccination sites because our objective was to measure the likelihood of existing healthcare facilities serving as COVID-19 vaccine administration locations. Nevertheless, we adjusted for the density of alternative vaccination locations. In other words, we accounted for the fact that healthcare facilities in counties heavily relying on non-traditional settings may have been less likely to administer COVID-19 vaccines.”

Results: Throughout the results presentation, the Ns for the number of pharmacies/vaccine sites should be presented in addition to the percentages. Without a sense of the number of eligible pharmacies/vaccine sites, the data are difficult to interpret. The data presentation would be strengthened by the ability to verify the vaccine distribution sites (a link to these publicly available data).

We have added the sample sizes for facilities in the first sentence of the results section and in the Table. We have included the link to CDC VaccineFinder data as a reference.

Discussion: The limitations of the data should be described in greater detail as indicated in the methods section of this review.

We have described the limitations of the data in the third and fourth paragraphs of the discussion section: “Our analyses were conducted at the facility level, but covariates were defined at the county level for two reasons: first, counties are the political unit with most control over local vaccine distribution; second, facility service areas can span hundreds of census tracts. Our sample did not include non-traditional vaccination sites because our objective was to measure the likelihood of existing healthcare facilities serving as COVID-19 vaccine administration locations. Nevertheless, we adjusted for the density of alternative vaccination locations. In other words, we accounted for the fact that healthcare facilities in counties heavily relying on non-traditional settings may have been less likely to administer COVID-19 vaccines. Our analyses are based on data from May 2021, and thus represent the early distribution of COVID-19 vaccines, when every US adult became eligible for vaccination. Future studies should reproduce analyses to test whether disparities have persisted over time. Our evaluation of racial/ethnic disparities focused on Non-Hispanic Black and Hispanic due to the low proportion of other minority groups in most counties, which yielded unstable estimates.

Several limitations of our study are related to the data sources, and particularly to the use of CDC VaccineFinder data for the identification identify actual vaccine administration locations. First, locations not registered in VaccineFinder were not identified, which could have led to an underestimation of the proportion of healthcare facilities serving as vaccine administration locations. Second, CDC VaccineFinder did not have information on the number of vaccines delivered in each site. As a result, our outcome was defined as a binary variable (whether a facility served as a COVID-19 vaccine administration site), and we were not able to measure the number of vaccines delivered in each facility. Third, we did not evaluate variation in the types of vaccines distributed to each healthcare facility. Fourth, the data compiled only contained information on the addresses of vaccine administration sites, so it was not possible to differentiate whether facilities did not serve as COVID-19 administration sites because of institutional or structural reasons. Fifth, our data and analyses were at the facility level rather than at the person level so we were not able to assess individual preferences in accessing healthcare facilities for vaccination”.

**References**
