# Supplementary material for: Disparities in distribution of COVID-19 vaccines across US counties: A geographic information system–based cross-sectional study
Source: PLoS Med. 2022 Jul 28;19(7):e1004069. doi: 10.1371/journal.pmed.1004069 (PMC9333439; doi:10.1371/journal.pmed.1004069)
Supplement: Attachment — Submitted filename: responses.doc [file pmed.1004069.s004.doc]

**Comments from the reviewers:**

**Reviewer #1:** The authors have responded to each comment in turn, and have suitably included social vulnerability index into the analysis.

Thank you.

**Reviewer #2:** Thanks to the authors for addressing my comments and those of others. Some of them may have seemed exasperating, but I think this version is clearer for the adjustments.

Line Edits:

Line 81, Should White be non-Hispanic White? "among racial/ethnic minority groups than White individuals" This occurs again later. It's fine to use short-hand after first use, particularly if you spell that out, like you do for "Blacks" being "non-Hispanic Blacks," but if there is ambiguity, I'd at least define it the first time. You could check all your race references for this. For instance, I might explain blacks again when first mentioned in the discussion. Line 218 could use some precision for White and Black too. Or change throughout so that "Black" and "White" never stand alone (and consider defining what Hispanic means?).

We appreciate this comment, we have now changed the text accordingly.

Line 105-106.When you are talking about LTCFs and how they aren't included, you could talk about the other types (like stadiums) that are not included? Perhaps move up Line 159 -165.

We have now clarified in line 103 that the sample included health care facilities only. We have added a sentence to lines 107-108 to acknowledge that non conventional vaccination sites were not included: “In a prior analysis, we identified and mapped open-door health care facilities eligible for the delivery of COVID-19 vaccinations”; “Non-traditional settings for vaccination such as stadiums or convention centers were not included since they are not health care facilities.”

Line 113. Add the month and year that adults got access to the vaccine.

We have made the requested change: “These data capture the healthcare facilities involved in the early distribution of COVID-19 vaccines, since the federal government required states to make vaccines available to the general public by May 1, 2021”.

Line 156-157 Rather than fixating on one of the parties, you could say you created a variable that said whether Trump or Biden won the election, unless there was someplace another candidate one. This would keep the audience from reading between the lines and getting tangled up in politics of COVID-19 and health equity.

We appreciate this suggestion, we have changed the text accordingly: “We created an indicator variable representing the Presidential candidate that won the 2020 election in each county.”

Line 172 I try to avoid sentences that start with dependent "because" clauses.

We have made the requested change: “We constructed one set of analyses for all four types of healthcare facilities and a second set for pharmacies because some FQHCs, RHCs, and HODs only offered COVID-19 vaccines to registered patients.”

Line 212 comma after metropolitan (trying to leave the line-editing to the line editor, sorry). Line 214 needs a comma after rural too.

We appreciate the editorial suggestions from the reviewer; they are very helpful to learn the editing of scientific writing. We have made the requested changes. We thank the reviewer for their time pointing out these suggestions to us.

Line 245 "Non-Hispanic Black and Hispanic" These could use a noun, like "people" or "minority groups."

We agree with the editorial suggestion, and we have changed the text accordingly: “Our evaluation of racial/ethnic disparities focused on Non-Hispanic Black and Hispanic individuals”

Line 248 identification identify

We apologize for the typo, we have now corrected the text.

Line 251 delivered "at" each site. Same issue for line 253, up to you.

We have made the suggested changes, we agree “at” is the correct preposition to use.

Line 255 suggested adjustment: "so it was impossible to define whether institutional or structural reasons led some facilities to not become COVID-19 administration sites". Sentences that lead with negatives always sound a bit awkward, but I don't how to fully remove it here.

We have incorporated the wording suggested by the reviewer.

Line 258 level","

We added the comma.

Line 259-262. This sentence could use a rewrite. Too many clauses, too many words.

We have split the sentence in two to improve clarity: “Our study however presents important strengths, including the use of spatial matching methods and the national coverage of the results. The innovative approach used to measure health equity should also be noted, since we used facilities as observations, instead of individuals or census tracts.”

Line 263 The discussion "regarding the causes of" lower uptake. I hope the discussion isn't focused on mistrust. Makes peer review more difficult, if so.

We appreciate the reviewer concern. Our analysis suggests that systematic barriers play an important role in differential rates of COVID-19 vaccination across racial/ethnic groups, which is often omitted in conversations around mistrust. We have updated the text accordingly.

Line 265 starts with almost a pun, I like it.

We appreciate the comment.

Line 266 Does this journal allow references in the middle of sentences? if so, never mind.

We are not sure about the journal guidance. We defer to the editorial team whether the reference should be moved to the end of the sentence.

**Reviewer #3:** The authors responded adequately to many of the reviewers' comments, and specifically to many of my own previous comments. However, two remaining glaring concerns are as follows:

1. Is it correct to draw conclusions about (and quantify) vaccine equity based only on number of sites eligible vs number of sites that actually distributed vaccines without the context of how many vaccines were distributed? Eg, even if 10 sites in a given area are eligible to distribute vaccines but only 5 of the sites actually distribute the vaccines, as long as those 5 sites distribute enough vaccines to the area's population then equity is achieved despite the fact that all 10 eligible sites did not distribute vaccines. As the authors used county-level measures for race/urbanicity, etc, why not additionally evaluate county level measures for number of COVID vaccines distributed during the study period? These county level data certainly exist, though they may exist outside the vaccine finder dataset.

States generally report data on the number of vaccines administered in each county, but not on the number of vaccines *shipped* to each county. The objective of our analysis is to measure equity in the *distribution* of COVID-19 vaccines. The number of vaccines administered in each county is not a good measure of equity in the distribution process because it is confounded by rates of vaccination uptake and by individuals crossing county lines to get vaccinated. In other words, it is not possible to differentiate whether a lower number of vaccines administered in a county is due to a lower number of vaccines shipped to that county or a lower willingness of the population to get vaccinated. For this reason, we have not incorporated the suggested analyses.

2. The authors have made clear that they are unable to compare data at multiple pandemic time points, though the reason why they are unable to extract data for later pandemic time points is unclear (it should be available from the same sources the authors used to evaluate the May 2020 time point, eg from CDC's vaccine finder). Longitudinal evaluation of the data that the authors have put forward is essential to determine whether geographic disparity in vaccine distribution existed during the pandemic and whether or not it improved. Given the number of unknown variables that may have influenced the authors' finding that all eligible vaccine distribution sites did not distribute vaccines early in the pandemic, the utility of this stand-alone/cross sectional finding is unclear. If this finding persisted over time--particularly in the context of comparatively fewer vaccines being distributed to underserved populations living in a given county over time--a more meaningful/impactful conclusion could be drawn.

We respectfully disagree with the reviewer comment that longitudinal evaluation of the data is essential to determine whether geographic disparity in vaccine distribution existed during the pandemic. Our findings of inequitable distribution can help guide future vaccine rollout efforts by highlighting the need for equity to be considered at the start of vaccine distribution process. Because Black populations were suffering higher infection rates and mortality from COVID in May 2020 than White populations, our finding of inequitable distribution holds important lessons for the prioritization of equity in initial pandemic response.

Below we summarize the reasons why we have not updated analyses with more recent data.

First, equity in the distribution of vaccines implies that vaccines were distributed in a timely manner across all individuals (if vaccines were distributed first to predominantly White neighborhoods and later on to underrepresented neighborhoods, the distribution process would be inequitable). The relevance of the timing of distribution is the reason why our data based on the month when vaccines first became available to the overall population are the most relevant in the assessment of equity in the vaccine distribution process. This is summarized in a new paragraph in the discussion section (lines 247-252).

Second, we do not think we can obtain appropriate data for longitudinal analyses. The data from actual COVID vaccination sites was obtained from CDC vaccine finder data. CDC vaccine finder has updated data, but is unclear whether results using May 2022 data would be representative at all, since it represents one year after wide availability of vaccination. The CDC vaccine finder data do not include a variable for when each facility started supplying vaccines, so it would not be accurate to perform analyses for intermediate time points.

Third, our analyses are based on geocoding and spatial matching methods that cannot be executed within the short time frame allowed for revision. The address data underwent an extensive analytical process to be standardized, geocoded, and then spatially matched using two independent methods, as outlined in the manuscript. This process was completed over a span of three months.
