# Supplementary material for: Disparities in distribution of COVID-19 vaccines across US counties: A geographic information system–based cross-sectional study
Source: PLoS Med. 2022 Jul 28;19(7):e1004069. doi: 10.1371/journal.pmed.1004069 (PMC9333439; doi:10.1371/journal.pmed.1004069)
Supplement: Attachment — Submitted filename: response to comments.docx [file pmed.1004069.s005.docx]

**Requests from Editors:**

1)	Please temper claims of primacy in the discussion (i.e., “… because it is the first to analyze equity in the distribution…”) by stating, "to our knowledge" or something similar.

Thank you for the comment, we have incorporated the suggested change: “Nevertheless, our analysis is a major contribution to the existing literature because, to our knowledge, it is the first to analyze equity in the distribution of vaccines to healthcare facilities across the nation”.

**Notes from Academic Editor:**

I think the reviewers' remaining (reviewer #3) concerns are justified and I also think the authors’ responses are reasonable. Nevertheless, I do think the authors should acknowledge some of these issues in their limitations.

Please see below. We have acknowledged the limitations highlighted by reviewer #3 in our manuscript.

On my read, the reviewer is questioning in part how meaningful the fraction of eligible facility delivering vaccines is, because this does not account for the volume of vaccines given per site, and a geography could be “covered” even if fewer facilities offered if those that did offered enough and were accessible enough. The authors respond that they are interested in the “distribution of vaccines” — I find this reasonable but not entirely convincing for two reasons. First, they say that whether a facility distributes at all is a more meaningful indication of equity in the supply, but it is not clear to me that regional demand does not influence whether a facility distributes at all. In other words, do we know that distribution as has been measured here is not influenced by demand? I do, nevertheless agree, that the “distributed or not” outcome is still valuable.

We have added a new paragraph in the discussion section to acknowledge these limitations: “Our nationwide quantitative evaluation of health equity in the early distribution of COVID-19 vaccines is limited by the data available. We operationalized the definition of health inequity as differences in the likelihood of a health care facility administering vaccines associated with the sociodemographics of the population served. This binary outcome does not account for important variables not available in the data such as the number of vaccines distributed in each health care facility, the volume of vaccines distributed in other facilities within the county, or the area of service of each facility. One could argue that the likelihood of a facility administering vaccines is not relevant because an area could be well covered by a smaller fraction of facilities, if such facilities provided enough vaccines to cover the population and were accessible enough. Nevertheless, regardless of whether a smaller share of facilities is able to adequately cover the population through higher volume, the variation in the proportion of available health care facilities used associated with the sociodemographic characteristics of the population suggests structural inequities in the design of the early COVID-19 vaccine rollout. The differential use of available health care infrastructure by the demographics of the population served may suggest prioritization of convenience for some residents rather than the actual distribution volume needed.”

Second, I do think that the authors need to be clear that they are looking at a particular moment in time. The equity of distribution of COVID related services (whether testing or vaccines) changed dramatically over time. Regardless of how it ended up, the initial geographical distribution of vaccine supply is important, but it should be noted if this was during a particular swath of time. May 10, 2021 was very early in the vaccine roll out. In fact the whole paper needs to be clear that this is disparities in the initial roll out.

We have noted throughout the manuscript that our analysis examines disparities in the early distribution of the vaccine roll-out process. We have clearly stated in the abstract and discussion that our findings may not be generalizable to later phases of the vaccine distribution process.

- Abstract:
  - “ The objective our study was to measure equity in the **early distribution** of COVID-19 vaccines to healthcare facilities across the US”
  - “We tested whether the likelihood of a healthcare facility administering COVID-19 vaccines **in May 2021** differed by county-level racial composition and degree of urbanicity”
  - “The outcome was whether an eligible vaccination facility actually administered COVID-19 vaccines **as of May 2021**”
  - “Across the US, 61.4% of eligible healthcare facilities and 76.0% of eligible pharmacies provided COVID-19 vaccinations **as of May 2021**”
  - “Our analyses did not include non-traditional vaccination sites and are based on data as of May 2021, thus **they represent the early distribution of COVID-19 vaccines**”
  - “Our results based on this cross-sectional analysis may **not be generalizable to later phases of** the COVID-19 vaccine distribution process”
  - “Healthcare facilities in counties with higher Black composition, in rural areas, and in hardest-hit communities were less likely to serve as COVID-19 vaccine administration locations **in May 2021**”.
- Author summary:
  - “We tested whether healthcare facilities serving minority or disadvantaged neighborhoods were less likely to administer COVID-19 vaccines in **the early phase** of the COVID-19 vaccine roll-out process”
  - “We found that healthcare facilities in counties with higher Black composition, in rural areas, and in hardest-hit communities were less likely to administer COVID-19 vaccines **in** **May 2021”**.
  - “There were disparities in **the early distribution** of COVID-19 vaccines to healthcare facilities across the country”.
- Introduction:
  - “To answer this question, we tested whether the likelihood of an eligible healthcare facility administering COVID-19 vaccines **in May 2021** varied with the county-level racial/ethnic composition and degree of urbanicity”
  - “This approach enabled us to quantify equity in the **early distribution** of COVID-19 vaccines to facilities.”
- Methods:
  - “These data capture the healthcare facilities involved in the **early distribution** of COVID-19 vaccines, since the federal government required states to make vaccines available to the general public by May 1, 2021”
  - “The outcome was whether an eligible vaccination facility was registered as actually administering COVID-19 vaccines to the public **in May 2021”**
- Results:
  - “Across the US, 61.4% of eligible healthcare facilities and 76.0% of eligible pharmacies provided COVID-19 vaccinations **in May 2021**”
  - “**In the early phase of the vaccine roll-out process**, healthcare facilities were less likely to serve as COVID-19 vaccine administration locations when located in counties with …”
- Discussion:
  - “To our knowledge, we present the first nationwide study to quantify disparities in **the** **early distribution of COVID-19 vaccines** to healthcare facilities in the US”
  - “Our study demonstrates that healthcare facilities in counties with higher non-Hispanic Black composition, in rural areas, and in hardest-hit communities were less likely to serve as COVID-19 vaccine administration locations **in May 2021”**
  - **“Our findings based on May 2021 data represent the early distribution of COVID-19 vaccines and may not be generalizable to later phases of the COVID-19 vaccine roll-out process”**
  - “We demonstrate that facilities in counties with higher Non-Hispanic Black composition, in rural areas, and in hardest-hit communities were less likely to serve as COVID-19 vaccine administration locations **in the early phase of the vaccine roll-out process”**
  - **“**We demonstrate that underrepresented, underserved, and rural areas have lower access to COVID-19 vaccines not only because of the lower concentration of healthcare facilities, but also because the facilities serving these areas were less likely to administer COVID-19 vaccines, **at least in the early phase of the vaccine distribution process**”
  - **“**The association between vaccine distribution and sociodemographics of the population served by facilities **could have changed over time**. In other words, our **findings based on May 2021 data may not be generalizable to later phases of the COVID-19 vaccine roll-out process”**
  - **“**the facilities that serve these vulnerable populations were less likely to administer COVID-19 vaccines **in the early phase of the vaccine roll-out process”**
  - “public health authorities should review COVID-19 distribution plans to identify the reasons why these processes resulted in an inequitable distribution of COVID-19 vaccines **in early 2021**”
  - “We evaluated whether the likelihood of healthcare facilities serving as COVID-19 vaccine administration sites in **the early distribution of COVID-19 vaccines** varied with the sociodemographic composition of the population served”
  - “We found that healthcare facilities in counties with higher Black composition, in rural areas, and in hardest-hit communities were less likely to serve as COVID-19 vaccine administration locations in **the early phase of the vaccine roll-out process”.**
